# AZD1480 Blocks Growth and Tumorigenesis of RET- Activated Thyroid Cancer Cell Lines

**DOI:** 10.1371/journal.pone.0046869

**Published:** 2012-10-02

**Authors:** Joana P. Couto, Ana Almeida, Laura Daly, Manuel Sobrinho-Simões, Jacqueline F. Bromberg, Paula Soares

**Affiliations:** 1 Institute of Molecular Pathology and Immunology of the University of Porto (IPATIMUP), Cancer Biology, Porto, Portugal; 2 Faculty of Medicine of the University of Porto, Porto, Portugal; 3 Institute of Biomedical Sciences Abel Salazar of the University of Porto, Porto, Portugal; 4 Department of Medicine, Memorial Sloan-Kettering Cancer Center, New York, New York, United States of America; 5 Department of Pathology, Hospital São João, Porto, Portugal; 6 Department of Medicine, Weill Cornell Medical College, New York, New York, United States of America; Consiglio Nazionale delle Ricerche (CNR), Italy

## Abstract

Persistent RET activation is a frequent event in papillary thyroid carcinoma (PTC) and medullary thyroid carcinoma (MTC). In these cancers, RET activates the ERK/MAPK, the PI3K/AKT/mTOR and the JAK/STAT3 pathways. Here, we tested the efficacy of a JAK1/2- inhibitor, AZD1480, in the *in vitro* and *in vivo* growth of thyroid cancer cell lines expressing oncogenic RET. Thyroid cancer cell lines harboring *RET*/PTC1 (TPC-1), *RET* M918T (MZ-CRC1) and *RET* C634W (TT) alterations, as well as TPC-1 xenografts, were treated with JAK inhibitor, AZD1480. This inhibitor led to growth inhibition and/or apoptosis of the thyroid cancer cell lines *in vitro*, as well as to tumor regression of TPC-1 xenografts, where it efficiently blocked STAT3 activation in tumor and stromal cells. This inhibition was associated with decreased proliferation, decreased blood vessel density, coupled with increased necrosis. However, AZD1480 repressed the growth of STAT3- deficient TPC-1 cells *in vitro* and *in vivo*, demonstrating that its effects in this cell line were independent of STAT3 in the tumor cells. In all cell lines, the JAK inhibitor reduced phospho-Y1062 RET levels, and mTOR effector phospho-S6, while JAK1/2 downregulation by siRNA did not affect cell growth nor RET and S6 activation. In conclusion, AZD1480 effectively blocks proliferation and tumor growth of activated RET- thyroid cancer cell lines, likely through direct RET inhibition in cancer cells as well as by modulation of the microenvironment (e.g. via JAK/phospho-STAT3 inhibition in endothelial cells). Thus, AZD1480 should be considered as a therapeutic agent for the treatment of RET- activated thyroid cancers.

## Introduction

The REarranged during Transfection (RET) proto-oncogene encodes for one of the first receptor tyrosine kinases (RTKs) that were found to be involved in cancer [1]. RET ligands belong to the glial cell- derived neurotrophic (GDNF) family and, upon engagement with RET, induce autophosphorylation of intracellular tyrosine residues, to which several adaptors bind [2]. These adaptors mediate the activation of multiple pathways, including the mitogen activated protein kinase (MAPK) signaling pathway, the phosphatidylinositol 3-kinase (PI3K) pathway, the c-jun N-terminal Kinase (JNK) pathway, the p38 pathway, SRC, ERK5, PLC-γ and Signal Transducer and Activator of Transcription (STAT)3 [3].

The first oncogenic role of RET was described in the most common endocrine cancer, papillary thyroid carcinoma (PTC) [4], as the result of genomic rearrangements leading to its constitutive activation and to increased cell survival, proliferation and motility [5]. *RET*/PTC rearrangements are the second most common genetic alteration in PTC, found in 30% of the cases [6]. *RET* point mutations were also found in medullary thyroid carcinoma (MTC) [7], [8], accounting for nearly all hereditary cases and about 50% of sporadic MTC [9].

Oncogenic RET has been implicated in mediating tumor-associated inflammation, as mutant forms of RET induced the expression of IL-8 [10] and other inflammatory molecules [11]. Furthermore, RET/PTC upregulated a set of inflammation- related genes in thyrocytes [12], [13] many of which belong to the IL-6/JAK/STAT3 pathway [14]. IL-6/JAK/STAT3 signaling is triggered by IL-6 coupling to its receptor-complex, comprising a receptor for IL-6 (IL-6R) and the signal transducing component, gp130 [15]. Subsequent phosphorylation of receptor-associated JAKs mediates tyrosine phosphorylation of STATs, particularly STAT3. Additionally, IL-6 activates the ERK/MAPK and PI3K/AKT pathways [16]. Deregulated JAK/STAT signaling (hyperactivation) has been described in a variety of diseases, including cancer [17]–[20]. Selective JAK1/2 small-molecule inhibitors that have been developed to treat JAK- mutated myeloproliferative disorders [21], [22] are currently in clinical trials for a variety of cancers. AZD1480 is a potent small-molecule JAK1/2 inhibitor [23] that is under phase I clinical trials for the treatment of myeloproliferative diseases. We investigated the effects of AZD1480 on IL-6/JAK and RET- dependent signaling (STAT3, ERK/MAPK and PI3K/AKT) as well as its biological effects in human thyroid cancer models (cell lines and a xenograft model). AZD1480 efficiently inhibited the growth and tumorigenesis of thyroid cancer cell lines harboring oncogenic *RET* alterations, likely through inhibition of PI3K/AKT signaling, supporting the use of this inhibitor for patients with thyroid cancer, particularly those with advanced MTC, for whom there are no effective therapeutic options.

## Results

### AZD1480 blocks the growth of thyroid cancer cell lines harboring *RET* oncogenic alterations

In this study, we determined the sensitivity of thyroid cancer cell lines harboring oncogenic forms of *RET* to JAK1/2 inhibitor, AZD1480. Specifically, we analyzed PTC-derived TPC-1 (*RET*/PTC1 rearrangement), MTC-derived MZ-CRC1 (*RET* M918T mutation) and TT (*RET* C634W mutation) cell lines. As comparison, we treated the same cell lines with a MEK1/2 inhibitor, AZD6244, which has been shown to have low efficacy in *RET*-mutated cells [24], in contrast to *BRAF*-mutated cells. In accordance, we used two other thyroid cancer cell lines, K1 (PTC-derived) and C643 (anaplastic thyroid carcinoma- derived) that harbor *BRAF*V600E and *HRAS*G13R mutations, respectively, as controls of AZD6244 efficacy. Cells were treated over 5 consecutive days with AZD6244, AZD1480 or a combination of both drugs, and cell density was determined.

**Figure 1:**
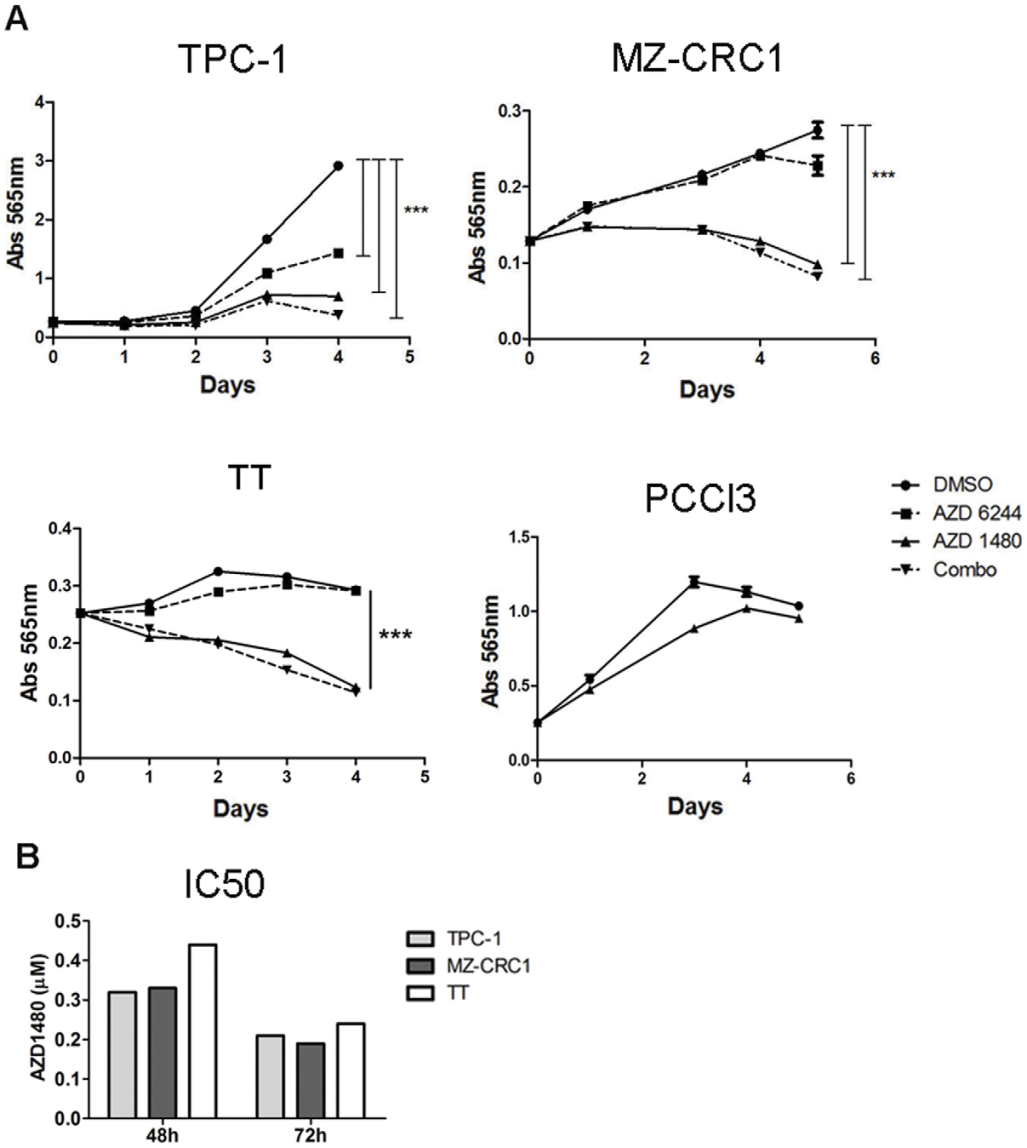
AZD1480 inhibits the growth of RET-activated thyroid cancer- derived cell lines. (A) TPC-1, MZ-CRC1 and TT cell lines were treated with AZD6244 (1 µM), AZD1480 (1 µM), and a combination of both drugs (1 µM each) for the indicated time. A rat- derived non malignant thyroid cell line, PCCl3, was treated with AZD1480 (1 µM) or control DMSO. Growth was determined by the sulphorhodamine B assay. (B) Cell lines were treated for 48 or 72 hours with different concentrations of AZD1480 (0.1, 0.5 and 1 µM) and cell density was determined. Data are shown as mean ± SE of three independent experiments. ***p<0.0001.

AZD1480 inhibited the growth of all *RET*-mutated/rearranged cell lines after 1 (MZ-CRC1, TT) and 2 days (TPC-1) of treatment (p<0.0001; Fig. 1A) and minimally decreased the growth of C643, while having no effect on K1 (Fig. S1). We observed that AZD6244 minimally decreased the growth of C643 and MZ-CRC1 after 4/5 days of treatment (p<0.001; Fig. 1A and S1), had no effect on the growth of TT (Fig. 1A) and decreased TPC-1 growth by 50% after 5 days of treatment. In contrast, AZD6244 efficiently inhibited the growth of *BRAF*- mutant K1 cell line (Fig. S1). No additive or synergistic effect of combined inhibition of MEKs and JAKs was observed. AZD1480 did not inhibit the growth of a non-malignant rat thyroid cell line, PCCl3 (Fig. 1A). The IC50's for AZD1480 were determined to be in the high nM range (∼200–450 nM) for these cell lines (Fig. 1B and S2), and decreased as a function of time (48 and 72 hours), suggesting a cytostatic effect (Fig. 1B).

**Figure 2:**
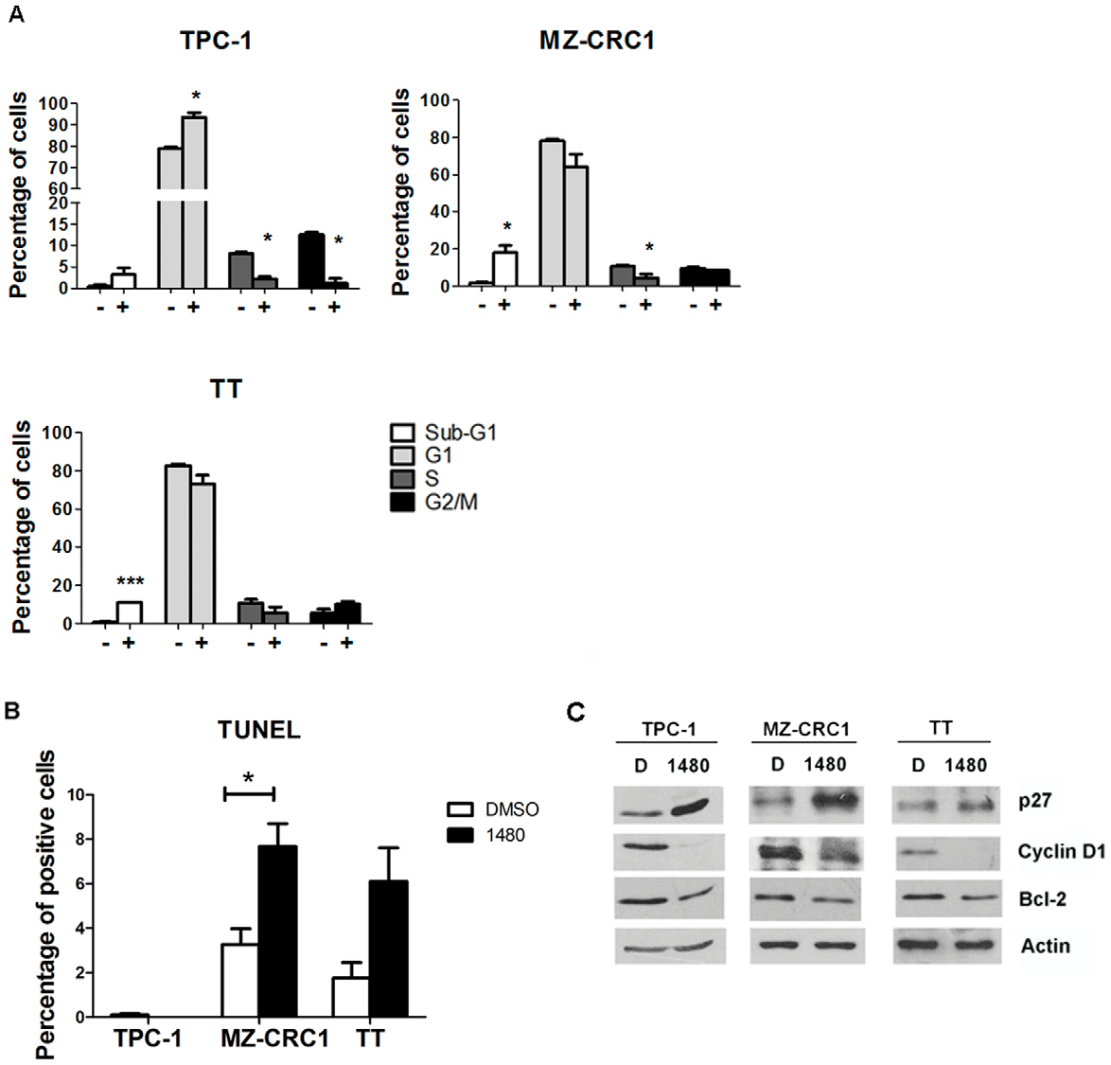
AZD1480 induces G1 blockage and apoptosis of thyroid cancer- derived cell lines. (A) TPC-1, MZ-CRC1 and TT were treated with AZD1480 (1 µM; +) for 72 hours. Cells were subjected to cycle profile analysis by flow cytometry. Results are representative of three independent experiments (mean ± SE). (B) Cells were treated with AZD1480 (1 µM) for 48 hours and apoptotic cell death was determined by TUNEL. (C) AZD1480 induces p27 upregulation and cyclin D1 and BCL-2 downregulation in thyroid cancer cell lines. TPC-1, MZ-CRC1 and TT cell lines were treated with AZD1480 (1 µM) for 24 hours. Cell lysates were probed with the indicated primary antibodies, by western-blotting. *p<0.05; ***p<0.0001.

Given the sensitivity of *RET*-mutated/rearranged cell lines to AZD1480, we further analyzed the cell cycle profile of TPC-1, MZ-CRC1 and TT treated for 72 hours with AZD1480 (Fig. 2A). The JAK inhibitor led to a G1 arrest in TPC-1 (94% compared with 79% in the control, p = 0.01). In the three cell lines, the percentage of cells in S-phase was decreased after AZD1480 treatment (TPC-1: 2% vs. 8% in the control, p = 0.01; MZ-CRC1: 4% vs. 11%, p = 0.004; TT: 6% vs. 11%, p = 0.09). Similarly, the proportion of cells in the G2/M was also decreased in TPC-1 cells treated with the JAK inhibitor (1% vs. 13% in the control, p = 0.01). In MZ-CRC1 and TT, a significant increase in the subG1 population (indicative of cell death by apoptosis) was detected (18% vs. 2%, in DMSO-treated, p = 0.04 and 11% vs 0.6%, p<0.0001, respectively) after 72 hours of AZD1480 treatment. To confirm the effect of AZD1480 in apoptosis, the cell lines were treated with AZD1480 for 48 hours and stained with TUNEL reagent, revealing an increase in the number of apoptotic MZ-CRC1 (p = 0.02) and TT (p = 0.1) cells compared to DMSO- treated cells (Fig. 2B). As expected, AZD1480 did not induce any apoptosis in TPC-1 (Fig. 2B). In parallel, we observed increased levels of the cyclin-dependent kinase inhibitor, p27, and decreased levels of cyclin D1 and of the anti-apoptotic protein, BCL-2, in AZD1480- treated cells (Fig. 2C).

### AZD1480 induces regression of TPC-1 xenografts

**Figure 3:**
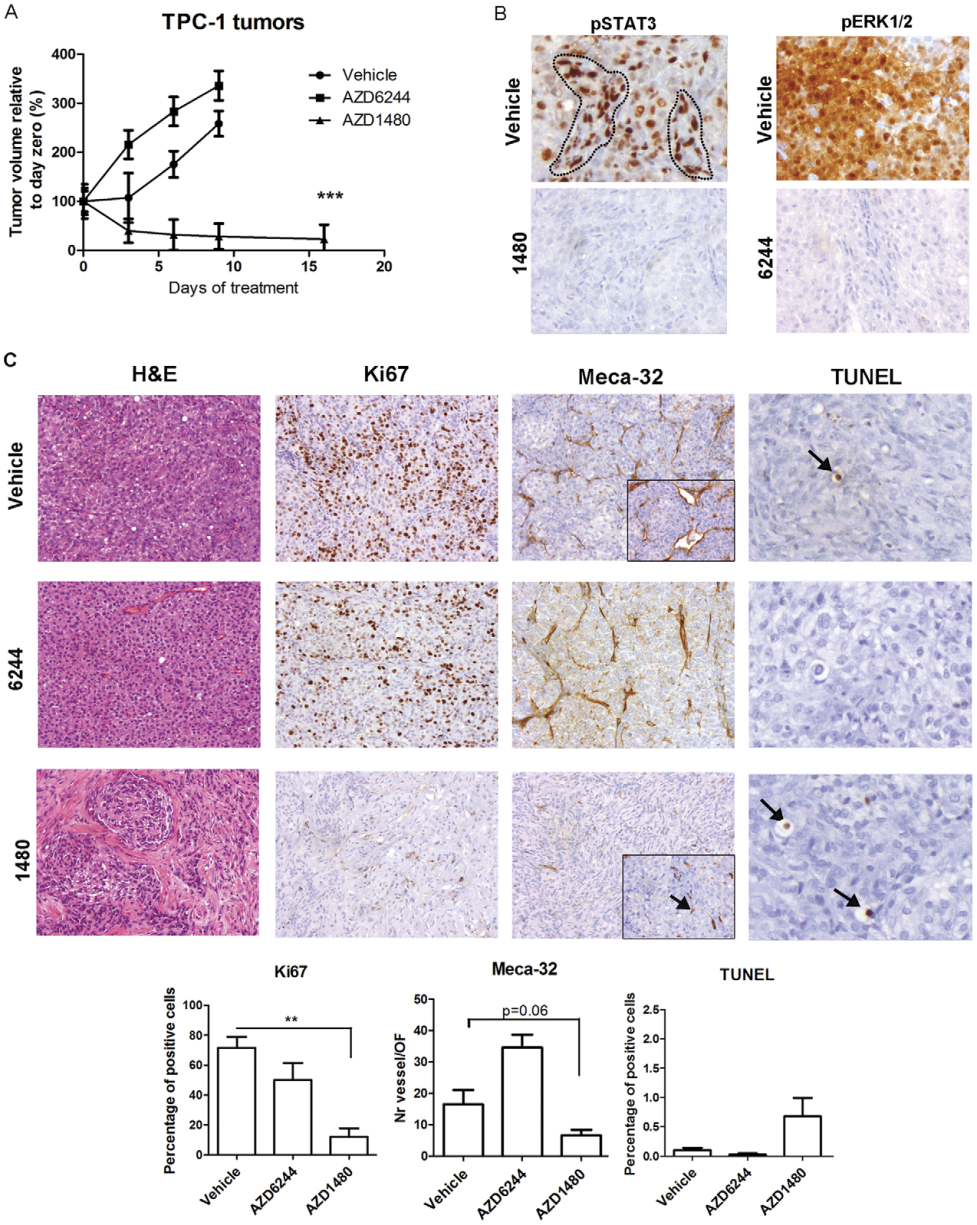
AZD1480 leads to TPC-1 xenograft tumor remission. (A) TPC-1 cells (10^6^) were injected subcutaneously in the flanks of nude mice. Following tumor engraftment (∼0.5 cm^3^), mice were distributed into four groups (n = 5/group) with similar mean tumor volumes. Groups were treated with drug vehicle (untreated), AZD6244, at 25 mg/Kg, once a day, or AZD1480, at 30 mg/Kg, bi-daily. Tumor volumes were determined bi-weekly (mean ± SE). (B) AZD1480- and AZD6244- treated TPC-1 tumor sections were immunostained for phospho-STAT3 and phospho-ERK1/2 expression, respectively. Phospho-STAT3-positive stromal cells are outlined with a dotted-line (magnification: 400x). (C) JAK inhibitor decreases proliferation and vasculogenesis of TPC-1 xenografts. H&E staining of TPC-1 tumors treated with drug vehicle (control), AZD6244 or AZD1480. Representative tumor sections were stained for proliferation (Ki67), angiogenesis (Meca-32) and apoptosis (TUNEL) markers. Original magnification: 200x. Quantification is represented graphically. **p<0.001; ***p<0.0001.

The effects of the JAK inhibitor on the *in vivo* growth of TPC-1 cells were evaluated by subcutaneous injection in the flanks of nude mice. When tumors reached ∼0.5 cm^3^, the mice were treated with vehicle, AZD1480 or AZD6244 for 16 consecutive days (Fig. 3A). The tumors from control mice and AZD6244- treated mice continued to grow until day 9 and due to their large size, the mice were sacrificed. In contrast, AZD1480- treated mice showed evidence of tumor regression after 4 days and, after 16 days, they measured ∼23% of their initial size (Fig. 3A). Immunohistochemical staining of representative tumor sections showed significant phospho-STAT3 downregulation by AZD1480 in tumor cells and stromal cells (endothelial cells). The MEK inhibitor, AZD6244 reduced phospho-ERK1/2 levels in tumors (Fig. 3B). Histologically, most of the tumor mass (90%) from AZD1480- treated tumors was composed of necrotic tissue, while the majority of tumors cells of the control and AZD6244 groups were viable and actively proliferating, as seen by Ki67 staining (Fig. 3C). Further characterization of these tumors revealed a reduction in endothelial cells (Meca-32) following AZD1480 treatment, compared to control and AZD6244 groups (p = 0.06 and p<0.0001, respectively; Fig. 3C). No significant differences were detected in the number of apoptotic cells (TUNEL), whose percentage was low throughout the tumors.

### AZD1480- mediated growth inhibition is independent of STAT3

**Figure 4:**
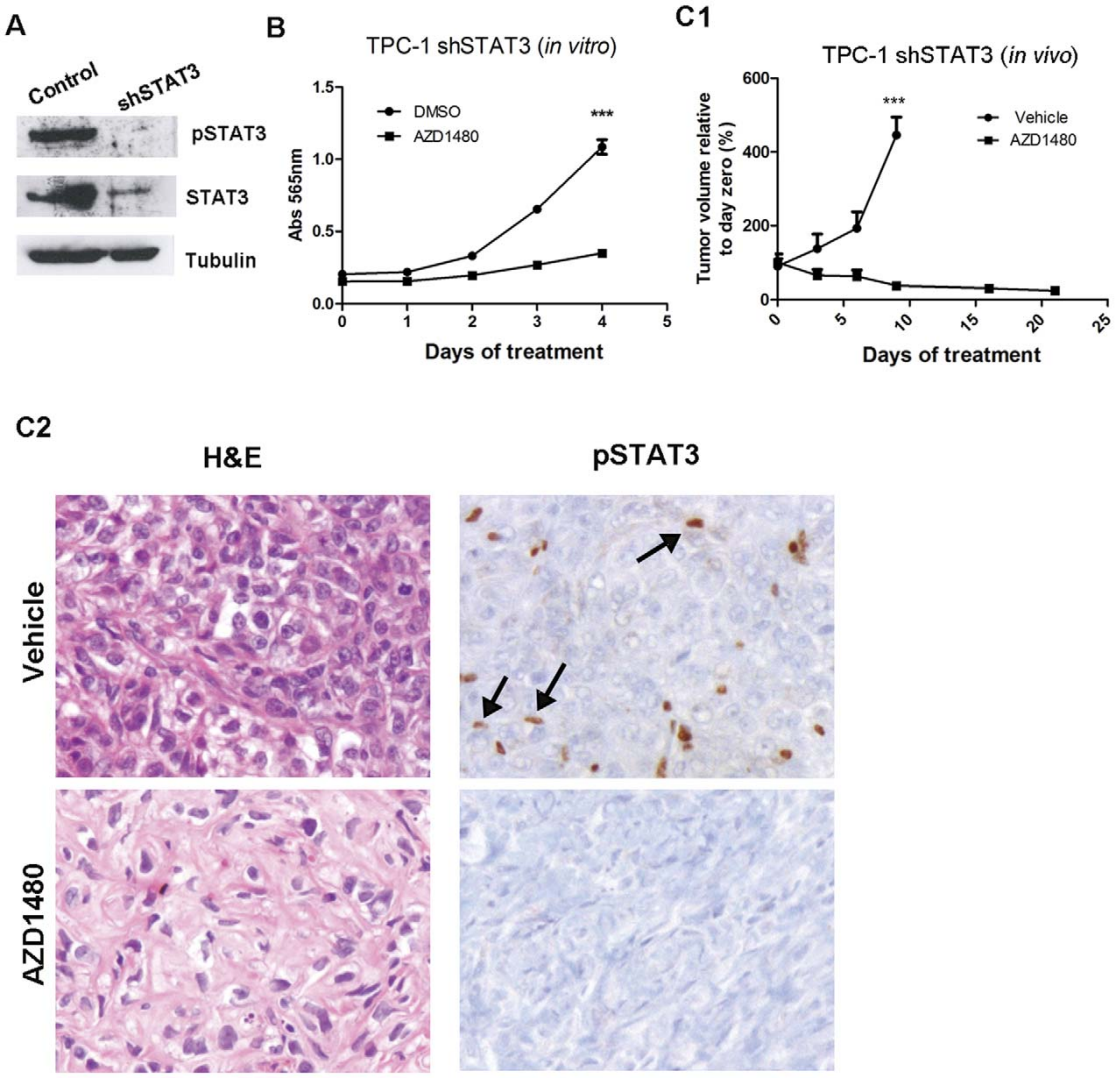
Growth inhibition of TPC-1 cells and tumors by AZD1480 is independent of activated phospho-STAT3 Tyr705. (A) STAT3 was knockdown in TPC-1 cells by a short hairpin. Phospho-STAT3 and STAT3 downregulation were confirmed by western-blotting. (B) TPC-1 shSTAT3 cells were treated with DMSO or AZD1480 (1 µM) and growth inhibition was determined by the SRB assay. Results are mean ± SE of three independent experiments. (C) TPC-1 shSTAT3 cells were injected subcutaneously in both flanks of nude mice. When tumors reached ∼0.5 cm^3^, mice were equally distributed in two groups: one was the control group (vehicle) and the other was treated with AZD1480 at 30 mg/Kg, bi-daily. (C1) Tumor volume was measured at the indicated time points. Results represent mean ± SE. (C2) Representative H&E and phospho-STAT3 immunostaining of tumors sections from untreated (vehicle) or AZD1480- treated TPC-1 shSTAT3 tumors. Arrows indicate phospho-STAT3- positive murine stromal cells. Magnification: 200x. ***p<0.0001.

JAKs are the principal mediators of IL-6/gp130/STAT3 signaling and, in several cancer models, JAK inhibitors' anti-tumorigenic effects are mediated by STAT3. In order to determine whether STAT3 was required for JAK inhibitor-mediated growth arrest, we stably reduced STAT3 in TPC-1 cells using a short hairpin, as determined by western-blot and immunohistochemistry (Fig. 4A, C2). Cells were treated with AZD1480 for four consecutive days and *in vitro* cell growth was monitored, revealing significant growth inhibition of the TPC-1 shSTAT3 cells (p<0.0001; Fig. 4B). *In vivo* growth was assessed by injecting the shSTAT3 cells subcutaneously and, upon reaching ∼0.5 cm^3^, tumor-bearing mice were treated with vehicle or AZD1480, for 21 days. The control group was sacrificed after 8 days due to the large size of the tumors. AZD1480 treatment induced regression (tumor volume was ∼24% of its initial size; p<0.0001) of TPC-1 shSTAT3 tumors (Fig. 4C1). Phospho-STAT3 was confirmed to be reduced in tumor cells of the vehicle- treated mice, but not in stromal cells, while tumor- and stromal- phospho-STAT3 were significantly reduced in AZD1480- treated mice (Fig. 4C2).

### AZD1480 inhibits RET Y1062 phosphorylation and downstream PI3K/AKT/mTOR signaling

**Figure 5:**
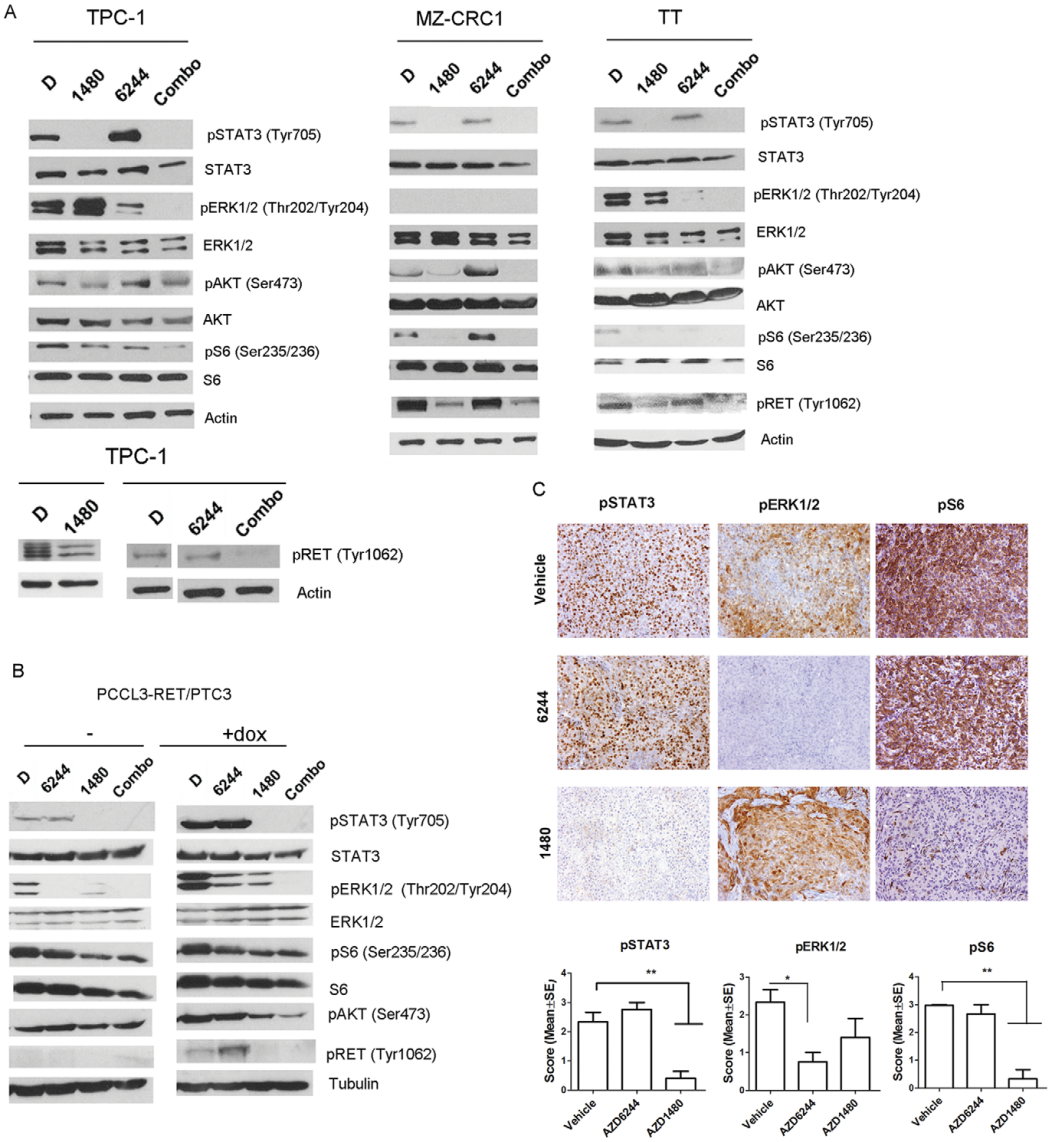
AZD1480 induces sustained downregulation of RET tyrosine phosphorylation and PI3K/AKT pathway activation *in vitro* and *in vivo*. (A) TPC-1, MZ-CRC1, TT and a (B) PCCl3-RET/PTC3 doxycycline-inducible cell line were treated for 24 hours with AZD1480 (1 µM), AZD6244 (1 µM), or a combination of both drugs. Total cell protein extracts were probed with antibodies for the indicated effectors of the JAK/STAT3, ERK/MAPK and PI3K/AKT signaling pathways as well as for phospho-RET Y1062. In the lower panel of TPC-1, the DMSO (D) lane is non-contiguous to the AZD6444 (6244) lane, in the same gel. (C) TPC-1 xenografts' representative sections were probed for the activation of STAT3 (phospho-STAT3), ERK/MAPK (phospho-ERK1/2) and PI3K/AKT (phospho-S6) signaling pathways. Quantification is represented graphically (mean ± SE). *p<0.05; **p<0.001.

**Figure 6:**
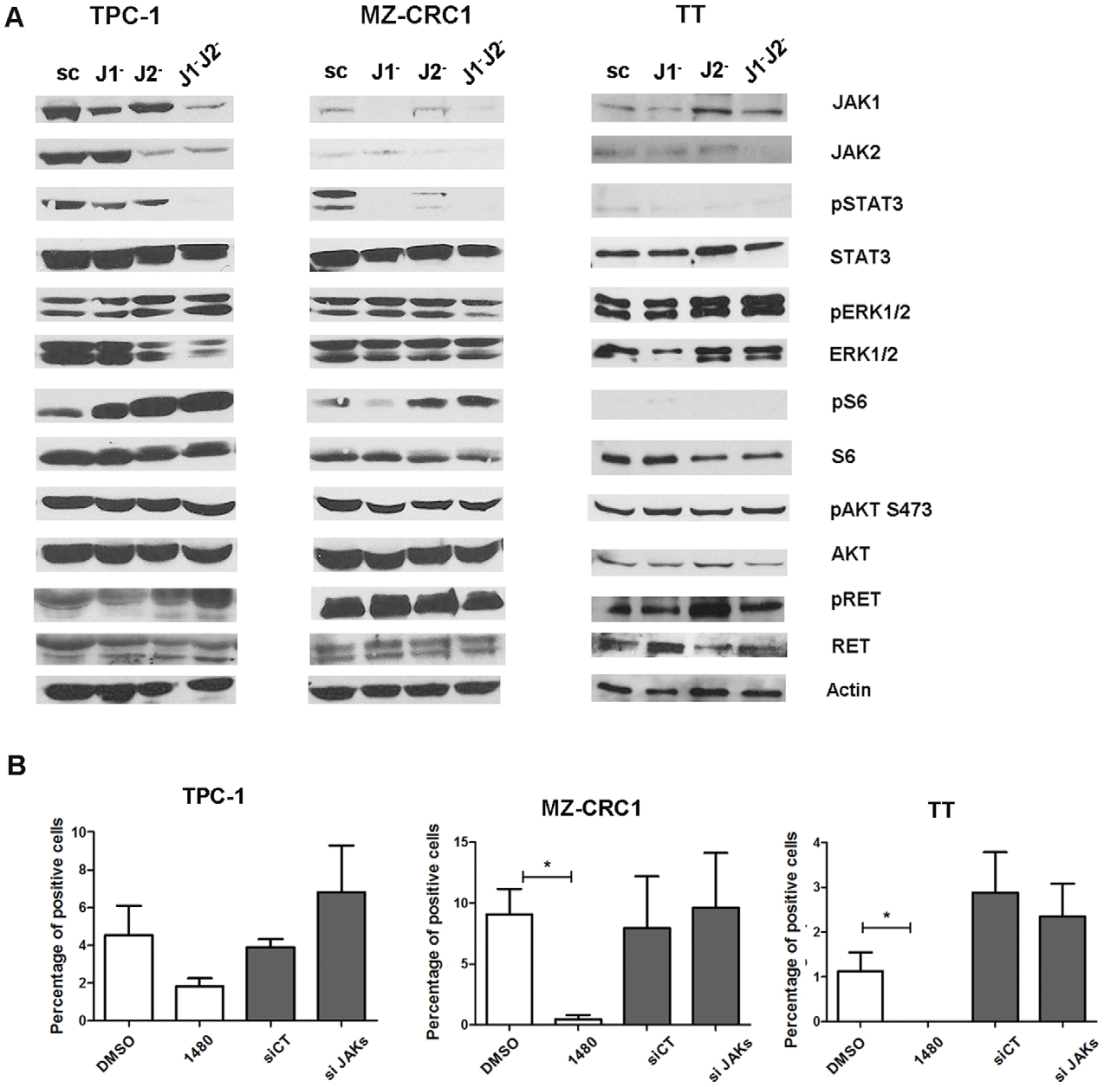
JAK1/2 knockdown by siRNA does not affect proliferation nor RET and PI3K pathway activation in thyroid cancer cell lines. (A) The expression of JAK1 or/and JAK2 was downregulated by siRNA in TPC-1, MZ-CRC1 and TT cell lines. The levels of the indicated proteins and their phosphorylation status were determined after 48 hours, by western-blot analysis. (B) Cell proliferation was measured by BrdU incorporation, 48 hours after transfection with a combination of siRNAs targeting both JAK1 and JAK2. Results represent mean ± SE of three independent experiments. *p<0.05.

Oncogenic RET effector pathways include ERK/MEK, PI3K/AKT and STAT3 [3]. Given the significant growth suppressive actions of the JAK inhibitor on the oncogenic *RET*- transformed TPC-1 xenograft independently of STAT3, we hypothesized that AZD1480 may have a direct effect on RET-mediated signaling. We treated TPC-1, MZ-CRC1, TT (Fig. 5A) as well as a model of inducible RET/PTC3 expression in PCCL3 (Fig. 5B), with AZD1480 and/or AZD6244, for 24 hours. The expression and phosphorylation levels of RET as well as of the main effectors of the JAK/STAT3, ERK/MAPK and PI3K/AKT pathway, namely phospho-STAT3 Tyr705, phospho-ERK1/2 Thr202/Tyr204 and phospho-AKT Ser473/phospho-S6 Ser235/236, respectively, were examined by western-blot analysis. AZD1480 and AZD6244 effectively decreased the levels of their downstream targets phospho-STAT3 and phospho-ERK1/2, respectively, in all of the cell lines (Fig. 5A, B). MZ-CRC1 did not express phospho-ERK1/2 at basal levels (Fig. 5A). Additionally, AZD1480 reduced the levels of phospho-ERK1/2 in PCCl3-RET/PTC3 and TT, as well as of phospho-AKT, phospho-S6 and phospho-RET in all of the cell lines (Fig. 5A, B). In contrast, AZD6244 treatment increased phospho-STAT3 in TPC-1 cells, increased phospho-AKT and phospho-S6 in MZ-CRC1 cells and increased phospho-RET in PCCl3-RET/PTC3 cells (Fig. 5A, B). There is no evidence to date demonstrating a functional association between RET and JAKs. To determine whether the decreased phospho-RET was due to off-target effects of AZD1480, we knockdown JAK1/2 expression in TPC-1, MZ-CRC1 and TT cells by short interfering (si) RNA. At 48 hours, JAK1, JAK2 and phospho-STAT3 levels were downregulated in all cell lines, with no effects on phospho-RET, phospho-ERK1/2 and phospho-S6 (Fig. 6A). In contrast to AZD1480, JAK1/2 knockdown did not decrease the proliferation of any of the cell lines, as seen by BrdU incorporation (Fig. 6B). We performed an in vitro kinase assay and verified that AZD1480 directly inhibited RET kinase activity: 40% inhibition at 0.001 μM, 90% inhibition at 0.1 μM and 99% at 1 μM (Fig. S3).

We examined the phosphorylation levels of STAT3, ERK and S6 in the TPC-1 xenografts treated with vehicle, AZD1480 and AZD6244. Similarly to what observed in TPC-1 and the other RET-activated cell lines *in vitro*, JAK inhibitor led to a significant decrease of phospho-S6 and phospho-STAT3 levels (p<0.001) in the tumor, while no decrease was observed in vehicle or AZD6244- treated tumors (Fig. 5C). Phospho-ERK levels were unchanged between the control and the AZD1480 groups and were significantly decreased (p = 0.01) in the AZD6244- treated group (Fig. 5C).

## Discussion

*RET* gene alterations are oncogenic “drivers” in thyroid cancer pathogenesis, particularly in MTC and PTC. Oncogenic RET is a potent activator of the ERK/MAPK and PI3K pathways and can induce the expression of inflammatory mediators such as CCL2, CXCL-1, GM-CSF, IL-1β and IL-6 [5], [25]–[27]. Moreover, RET/PTC and mutant RET can induce phosphorylation of STAT3 either directly [14], [28] or in a JAK-dependent manner [29]. JAKs are tyrosine kinases that mediate IL-6- dependent STAT3 activation, which has been shown to promote cancer progression in numerous examples of solid tumors. Importantly, JAK2 activating mutations are critical in the pathogenesis of myeloproliferative disorders and that has led to the development of JAKs small molecule inhibitors [22], [23]. Herein, we investigated the biological effects of a JAK1/2 inhibitor, AZD1480, on the growth of PTC- and MTC- derived thyroid cancer cell lines harboring activating *RET*/PTC rearrangements and *RET* mutations, respectively. We observed that AZD1480 inhibited the growth of TPC-1, MZ-CRC1 and TT with IC_50_s <500 nM, which is 2 to 10 fold below that reported for other cancer cell lines [23], [30]. The block in growth was due to a G1 cell cycle arrest in TPC-1 cells, while in MZ-CRC1 and TT, JAK inhibition significantly increased apoptosis. On the other hand, a MEK1/2 inhibitor, AZD6244, failed to alter *in vitro* growth of MZ-CRC1 and TT. No additive or synergistic effects on *in vitro* growth were observed by combining both inhibitors. On the contrary, AZD6244 (but not AZD1480) efficiently inhibited the growth of a *BRAF*V600E- mutant cell line, K1. Both AZD1480 and AZD6244 had a minimal effect on the growth of a *RAS*- mutant cell line, C643. The insensitivity of RET-activated thyroid cancer cells to MEK inhibition has been previously demonstrated, as opposed to the high sensitivity of thyroid cancer cells expressing *BRAFV600E* [31]. This resistance might reflect the ability of oncogenic RET to activate alternative signaling pathways, particularly the PI3K/AKT/mTOR pathway [32]. Moreover, AZD6244 caused upregulation of phospho-RET Y1062 in the PCCl3-RET/PTC3 model as well as of mTOR effectors, phospho-S6 and phospho-AKT, in MZ-CRC1. Overactivation of the mTOR pathway in response to MEK inhibition can possibly be explained by relief of feed-back inhibition and has been previously reported in other models [33], where it mediates cell resistance to AZD6244 [34], [35].

Furthermore, AZD1480 potently inhibited the *in vivo* growth of TPC-1 xenografts, resulting in tumor regression, while the tumors from AZD6244- treated mice grew slightly more than the control tumors, suggesting that treating *RET*-mutated thyroid cancers with this inhibitor may promote tumor growth. In TPC-1 tumors, and similarly to the effects *in vitro*, AZD1480 blocked the proliferation while not significantly affecting apoptosis. However, *in vivo*, we observed marked tumor regression, with tumors displaying extensive areas of necrotic tissue and a significant decrease in the number of blood vessels. The latter might have caused a drop in nutrient and oxygen supply to the tumors, likely explaining the accentuated necrosis and, consequently, tumor reduction. Such effects on tumor growth have been previously documented in other cancer models (lung, breast, prostate, brain) [23], [36], [37]. In these models, JAK inhibition was particularly effective in phospho-STAT3-positive tumors/cell lines. However, AZD1480 has also been shown to inhibit the growth of cancer cell lines independently of STAT3 activation, particularly at higher doses [23], possibly due to off-target effects of the drug. In a recent report, AZD1480 blocked both JAK/STAT3 and FGFR3 signaling in myeloma cells [30]. To test whether the growth inhibitory effects of AZD1480 were dependent on STAT3 in our models, we knocked-down STAT3 in TPC-1 cells. STAT3 deficiency in these cells did not affect their sensitivity to JAK inhibition as compared to control cells. Moreover, AZD1480 was similarly effective in blocking the growth of STAT3- deficient TPC-1 xenografts, which displayed extensive necrosis, similarly to AZD1480-treated parental TPC-1 tumors. Additionally, we knocked-down JAK1 and JAK2 expression in TPC-1, MZ-CRC1 and TT-cell lines by siRNA, which had no effect on their proliferation. These data demonstrate that AZD1480 inhibits the growth of RET-activated thyroid cancer cell lines *in vitro* and *in vivo*, independently of JAK/STAT3 signaling in cancer cells.

We sought to identify the mechanisms explaining the growth inhibitory effects of AZD1480 *in vitro* and *in vivo*. In all cell lines, AZD1480 efficiently reduced phospho-STAT3 levels, including the C634W mutant TT cell line, although this oncogenic form of RET was described as activating STAT3 independently of JAKs, through two docking sites on RET (Tyr752 and Tyr928) [28]. We suggest that our results differ due to the use of a different JAK inhibitor, with different potencies, than that used by Schuringa *et* *al*.

So far, no data have demonstrated a role for JAKs in RET activation (Y1062 phosphorylation) nor on activation of its downstream MAPK and PI3K pathways. We determined that AZD1480 blocked RET Y1062 phosphorylation in TPC-1, MZ-CRC1, TT, as well as in a conditional model of *RET/PTC3* expression. Moreover, although AZD1480 did not inhibit the ERK/MAPK pathway in most of our cell lines, it blocked the activation of the PI3K effectors AKT and S6. Similar results were obtained in the AZD1480- treated TPC-1 xenografts, where no differences in ERK/MAPK levels were detected, and phospho-S6 was significantly downregulated. We demonstrated that these effects were independent of JAKs, as phospho-RET, phospho-ERK and phospho-S6 levels did not change upon JAK1/2 knockdown by siRNA. We demonstrated that AZD1480 directly inhibits the kinase activity of recombinant RET in a dose-dependent manner, which likely underlines the inhibitory and mutant-RET specific effects of AZD1480 on the growth and survival of thyroid cancer cells. Indeed, *in vitro* kinase assays from a previous report have demonstrated that AZD1480 can inhibit ∼50% and 90% of RET activity at 0.1 and 1 µM concentrations, respectively [23].

In conclusion, we showed that the JAK1/2 inhibitor, AZD1480, can block the growth and induce cell death of thyroid cancer cell lines harboring distinct forms of oncogenic *RET in vitro* and *in vivo*. In these cells, AZD1480 likely inhibits RET directly, leading to the consequent blockade of the PI3K/AKT/mTOR pathway, which seems to be the preferential oncogenic force driving RET-activated cells. Although these effects were independent of STAT3 in thyroid cancer cells, AZD1480 effectively inhibited phospho-STAT3 in the stroma, particularly in endothelial cells. In fact, JAK inhibitors are known modulators of the microenvironment through inhibition of angiogenesis and myeloid cell mobilization in a STAT3- dependent manner [36], [38]. Given the significant decrease in the vascularity of AZD1480- treated tumors and consequent tumor necrosis, we suggest that phospho-STAT3 inhibition in the microenvironment (endothelial cells) cooperates with RET inhibition in cancer cells to induce tumor regression. Additionally, we cannot discard that other RET-independent tyrosine kinases (such as FLT1, FGFR [23]) may be affected by AZD1480, contributing to the growth arrest of RET- activated cells and tumors.

Importantly, MZ-CRC1, which harbors the M918 *RET* mutation associated with the MEN2B syndrome, was highly sensitive to the growth inhibitory effects of AZD1480. Patients diagnosed with MEN2B develop rapidly progressive, multifocal MTC with lymph node metastases, usually requiring total thyroidectomy before 1 year of age [39]. The greater sensitivity of MZ-CRC1 to AZD1480 compared with TT (harboring the less aggressive C634W mutation), might be explained by different capacities of MEN2A- and MEN2B- RET mutants to activate downstream pathways, namely the PI3K/AKT pathway [40]. Altogether, these results support the use of AZD1480 to treat aggressive forms of thyroid cancer, particularly MEN2B- MTC.

## Materials and Methods

### Ethics statement

All the procedures of animal research were included in a protocol (number 0011091) approved by the MSKCC Institutional Animal Care and Use Committee (IUAC), following the Laboratory Animals Welfare Act, the Guide for the Care and Use of Laboratory Animals and the Guidelines and Policies for Rodent experiment.

### Cell culture and drugs

TPC-1, K1, C643 (provided by Prof. Marc Mareel, Belgium) and TT (purchased from American Type Culture Collection – ATCC) [29], [41] cell lines were maintained in RPMI, 10% FBS, 1% PenStrep. PCCl3-RET/PTC3 was provided by Dr James Fagin and was cultured as previously described [13]. MZ-CRC1 (provided by Dr Robert Hofstra, Netherlands) [29] and 293T (ATCC) were maintained in DMEM, 10% FBS, 1% PenStrep. All cell culture reagents were from GIBCO, Invitrogen, Carlsbad, USA. AZD1480 [23] and AZD6244 [24] were gifts from Dennis Huszar and Michael Zinda (AstraZeneca, London, UK).

### Lentiviral infections and generation of stable cell lines

The STAT3 shRNA lentiviral construct was previously described [42]. Viral particles carrying the constructs were generated in 293T-cells. The viral particles in the supernatant were precipitated using a polyethylene glycol virus precipitation solution (System Biosciences LLC, Mountain View, CA, USA). Lentiviral constructs included a transcription cassette encoding enhanced green fluorescent protein [42], that allowed selection of cells by sorting.

### Whole cell protein extracts and western blotting

Cells were lysed in radio-immunoprecipitation assay buffer, supplemented with protease and phosphatase inhibitors (Sigma Aldrich, St. Louis, MO, USA). Proteins were quantified using a modified Bradford assay (Biorad Laboratories, Inc., Hercules, CA, USA), resolved by SDS-PAGE and transferred to Hybond ECL membranes (GE Healthcare, Little Chalfont, England). The primary antibodies for phospho-STAT3 (Tyr705), STAT3, phospho-ERK1/2 (Thr202/Tyr204), ERK1/2, phospho-AKT (Ser473), AKT, phospho-S6 (Ser235/236), and S6 were from Cell Signaling Technologies, Inc, Danvers, MA, USA. Cyclin D1 antibody was from Neomarkers, Fremont, CA, USA. Actin antibody, phospho-RET (Y1062), total RET (C-19) and p27 were from Santa Cruz BT, Santa Cruz, USA, BCL-2 antibody was from DAKO, Glostrup, Denmark, and α-tubulin was from Sigma Aldrich, St. Louis, MO, USA. Peroxidase- conjugated secondary antibodies were from Santa Cruz BT. Bands were visualized by chemiluminescence using ECL detection system (Perkin Elmer, Foster City, USA).

### *In vitro* cell growth

The cell density was assessed by sulforhodamine (SRB) staining, as previously described [43]. Cells were fixed with 10% trichloroacetic acid and stained with a 0.4% sulphorhodamine B (Sigma Aldrich, St. Louis, MO, USA) solution. Absorbances were read in a microplate reader, at 565 nm. The IC50s were determined by nonlinear regression using GraphPad Prism v5 (GraphPad Software Inc., San Diego, CA). Each experimental condition was performed 3 times, each including 6 replicates.

### Flow cytometry

For cell cycle analysis, cells were fixed with 70% ice-cold ethanol and stained with a solution containing 5 μg/ml propidium iodide (Sigma Aldrich, St. Louis, MO, USA) and 100 μg/ml RNase A (Fermentas, St Leon-Rot, Germany). The results were analyzed in FlowJo (Tree Star, OR, USA).

### Apoptosis assay

Cells were fixed with 4% paraformaldehyde at room temperature and permeabilized with 0.1% Triton X-100 in 0.1% sodium citrate (2 min) on ice. TdT mediated dUTP Nick End Labeling (TUNEL) was performed using the “In situ cell death detection kit, fluorescein” (Roche®), following the manufacturer's instructions. Each experimental condition was performed three times and the number of positive nuclei in a total of 500 cells was determined in a fluorescence microscope.

### JAK1 and JAK2 silencing

Pre-designed small interference RNAs (siRNAs) targeting human JAK1 (NM_002227) and JAK2 (NM_004972; NM_004972; NM_004972) were purchased from Qiagen, Hilden, Germany. The AllStars negative control with the following target sequence: 5′-AATTCTCCGAACGTGTCACGT-3′ was also from Qiagen. Cells were transfected with Lipogen (InvivoGen, Toulouse, France) and 25–50 nM of the siRNA. JAK1 and JAK2 down-regulation was verified after 48 hours.

### Proliferation assay

For analysis of cell proliferation, bromodeoxyuridine (BrdU) incorporation into cellular DNA during S-phase was determined. Cells were incubated with 10 μM bromodeoxyuridine (BrdU) for 1 hour, fixed with 4% paraformaldehyde, treated with HCl 2 M and incubated with a mouse anti-BrdU secondary antibody (DAKO, Glostrup, Denmark), followed by incubation with secondary anti-mouse antibody, conjugated with Alexa 594 (Invitrogen, Carlsbad, USA). Cells with nuclear positivity were counted in a total of 500 cells, under a fluorescence microscope.

### RET kinase assay

The kinase activity of RET was determined using 2 ng/μL of recombinant RET and 1 μg/μL of IGF1 as substrate, in the presence of ATP (50 μM), for one hour, at room temperature (Promega Corp., Madison, WI, USA). After ADP to ATP conversion, ATP was converted into light using the ADP-Glo Kinase Assay (Promega Corp.), according to manufacturer's instructions. The luminescent signal was recorded in a luminometer.

### *In vivo* tumorigenicity assay

Each experimental group included 5 female homozygous athymic nu/nu mice (Harlan Laboratories), all littermates. TPC-1 cells were harvested, mixed with an equal volume of matrigel (BD Biosciences, Franklin Lakes, NJ, USAhttp://en.wikipedia.org/wiki/United_States) and injected subcutaneously into the flanks of the mice. When tumors had approximately 0.5 cm^3^, mice were treated with the inhibitors. AZD6244 and AZD1480 were dissolved in 0.5% (wt/vol) hydroxyl-propyl-methylcellulose, 0.2% (vol/vol) Tween 80 (Sigma Aldrich, St. Louis, MO, USA). AZD6244 was administered once daily by oral gavage at a dose of 25 mg/kg. AZD1480 was administered by oral gavage bidaily, at 30 mg/kg. Tumor dimensions length (l) and width (w) were measured and tumor volume was calculated by the formula for ellipsoids *V*(tumor) = π*lw* ^2^/6 [44]. The animals were sacrificed by CO_2_ asphyxiation, following all the institutional procedures to avoid animal suffering. No signs of toxicity were detected during the drug treatments.

### Immunohistochemistry

Immunohistochemistry was carried out by the streptavidin-biotin-HRP method. Antigen retrieval was performed in citrate buffer (pH 6.0) (Ki67, phospho-ERK1/2) or Tris-EDTA (pH 9.0) (phospho-STAT3, phospho-S6) for 15 minutes, under boiling temperature, in a microwave. After peroxidase blocking in a 3% hydrogen peroxide solution and non-specific binding blocking using the Large Volume Ultra V Block reagent (Thermo Scientific/Lab Vision, Fremont, USA), samples were incubated with the respective primary antibodies (the same used in western-blotting). Tyramide Signal Amplification (TSA) Biotin System (Perkin-Elmer, Foster City, USA) was used for phospho-STAT3 signal amplification as well as for TUNEL (Roche Diagnostics Corp., Basel, Switzerland) staining, according to manufacturer's instructions. Ki67 RTU was from Novocastra (now Leica Microsystems, Wetzlar, Germany). Meca-32 was custom made by Developmental Studies Hybridoma Bank, IA, USA. As secondary reagents, we used a labeled streptavidin-biotin immunoperoxidase detection system (Thermo Scientific/Lab Vision, Fremont, USA) followed by DAB (3,3′-diaminobenzidine) developing.

The results were evaluated under a brightfield microscope and scored semiquantitatively (phospho-STAT3, phospho-S6, phospho-ERK1/2) or quantitatively (Ki67, Meca-32). For semiquantitative analysis, both the intensity (0, 1, 2, 3) and the percentage of positive cells (0–5%:1; 6–25%:2; 26–50%:3; 51–75%:4; 76–100%:5) were considered. The respective scores were multiplied and grouped in four classes: negative (score:0), low (+1; score 1–3), moderate (+2; score 4–8) and high (3+; score 9–15). For meca-32, the number of vessels was counted in at least 5 representative 400x optical fields. For Ki67 and TUNEL, at least 1000 cells were counted and the percentage of positive cells was determined.

### Statistical analysis

Statistical analysis was done in StatView (SAS Institute) and GraphPad, using t-Student test and ANOVA. Results were considered statistically significant when p<0.05 (*, p<0.05; **, p<0.001;***, p<0.0001).

## Supporting Information

Figure S1 **Sensitivity of** ***BRAF*** **and** ***RAS*** **- mutated thyroid cancer cell lines to JAK and MEK inhibition.** (A) K1 (*BRAF*V600E) and C643 (*HRAS*G13R) cell lines were treated with AZD6244 (1 µM), AZD1480 (1 µM), and a combination of both drugs (1 µM each) for the indicated time. Growth was determined by the SRB assay. ***p<0.0001.
(TIF)

Figure S2 **Dose-response curves from** ***RET*** **-mutated thyroid cancer cell lines treated with AZD1480.** Cell lines were treated with the indicated concentrations of the drug for 48 and 72 hours. Results represent mean ± SE of three independent experiments.
(TIF)

Figure S3 **AZD1480 inhibits RET kinase activity.** Recombinant RET was incubated with its substrate, IGF1, in the presence of ATP and DMSO (control) or different concentrations of AZD1480 (from 0.001 μM to 2 μM). The data represent percent activity of RET after compound treatment.
(TIF)
