# Supplementary figures and images for: AZD1480 Blocks Growth and Tumorigenesis of RET- Activated Thyroid Cancer Cell Lines
Source: PLoS One. 2012 Oct 2;7(10):e46869. doi: 10.1371/journal.pone.0046869 (PMC3462763; doi:10.1371/journal.pone.0046869)

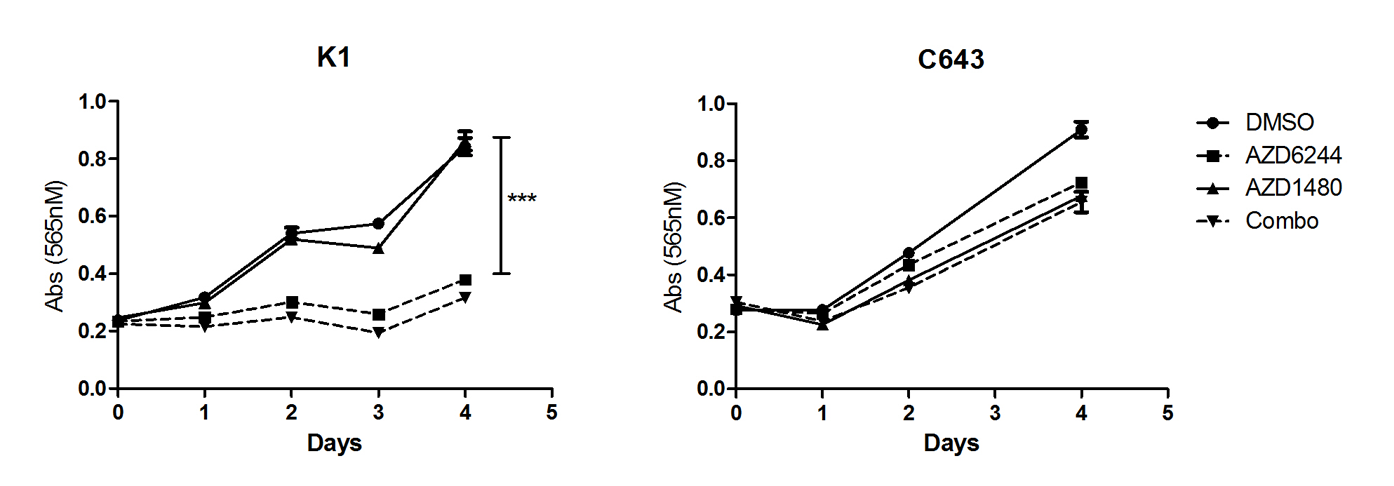

Supplement: Figure S1 — Sensitivity of BRAF and RAS - mutated thyroid cancer cell lines to JAK and MEK inhibition. (A) K1 (BRAFV600E) and C643 (HRASG13R) cell lines were treated with AZD6244 (1 µM), AZD1480 (1 µM), and a combination of both drugs (1 µM each) for the indicated time. Growth was determined by the SRB assay. ***p<0.0001. (TIF) [file pone.0046869.s001.tif]

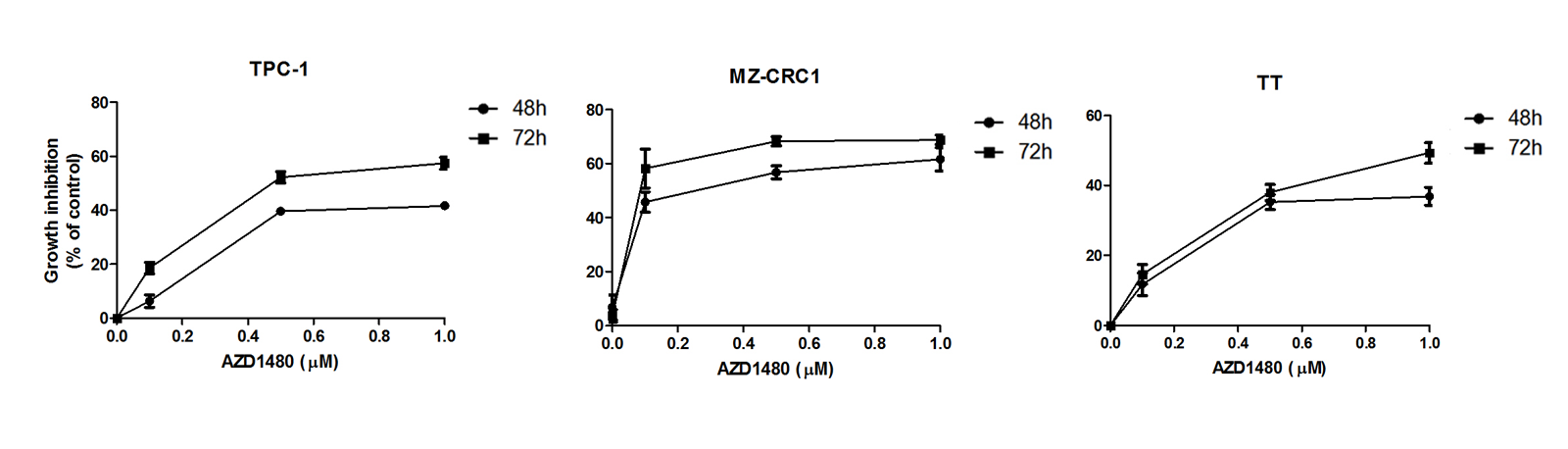

Supplement: Figure S2 — Dose-response curves from RET -mutated thyroid cancer cell lines treated with AZD1480. Cell lines were treated with the indicated concentrations of the drug for 48 and 72 hours. Results represent mean ± SE of three independent experiments. (TIF) [file pone.0046869.s002.tif]

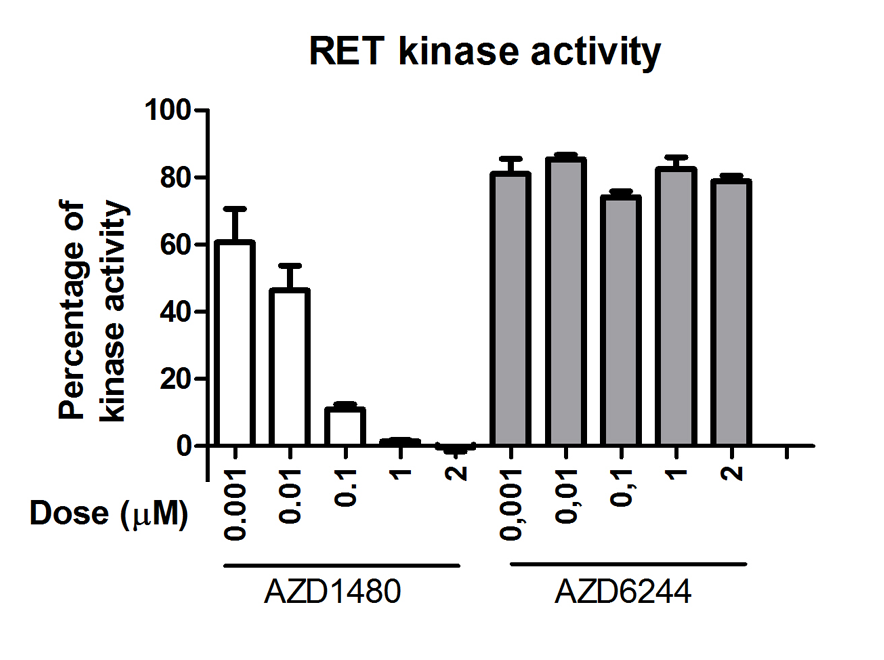

Supplement: Figure S3 — AZD1480 inhibits RET kinase activity. Recombinant RET was incubated with its substrate, IGF1, in the presence of ATP and DMSO (control) or different concentrations of AZD1480 (from 0.001 μM to 2 μM). The data represent percent activity of RET after compound treatment. (TIF) [file pone.0046869.s003.tif]
